# Deep Small RNA Sequencing Reveals Important miRNAs Related to Muscle Development and Intramuscular Fat Deposition in *Longissimus dorsi* Muscle From Different Goat Breeds

**DOI:** 10.3389/fvets.2022.911166

**Published:** 2022-06-13

**Authors:** Jiyuan Shen, Zhiyun Hao, Yuzhu Luo, Huimin Zhen, Yan Liu, Jiqing Wang, Jiang Hu, Xiu Liu, Shaobin Li, Zhidong Zhao, Yuan Liu, Shutong Yang, Longbin Wang

**Affiliations:** Gansu Key Laboratory of Herbivorous Animal Biotechnology, Faculty of Animal Science and Technology, Gansu Agricultural University, Lanzhou, China

**Keywords:** microRNA (miRNA), muscle development, intramuscular fat, small RNA sequencing, goat

## Abstract

MicroRNAs (miRNAs) are a class of small non-coding RNAs that have been shown to play important post-transcriptional regulatory roles in the growth and development of skeletal muscle tissues. However, limited research into the effect of miRNAs on muscle development in goats has been reported. In this study, Liaoning cashmere (LC) goats and Ziwuling black (ZB) goats with significant phenotype difference in meat production performance were selected and the difference in *Longissimus dorsi* muscle tissue expression profile of miRNAs between the two goat breeds was then compared using small RNA sequencing. A total of 1,623 miRNAs were identified in *Longissimus dorsi* muscle tissues of the two goat breeds, including 410 known caprine miRNAs, 928 known species-conserved miRNAs and 285 novel miRNAs. Of these, 1,142 were co-expressed in both breeds, while 230 and 251 miRNAs were only expressed in LC and ZB goats, respectively. Compared with ZB goats, 24 up-regulated miRNAs and 135 miRNAs down-regulated were screened in LC goats. A miRNA-mRNA interaction network showed that the differentially expressed miRNAs would target important functional genes associated with muscle development and intramuscular fat deposition. Kyoto Encyclopedia of Genes and Genomes (KEGG) enrichment analysis revealed that the target genes of differentially expressed miRNAs were significantly enriched in Ras, Rap 1, FoxO, and Hippo signaling pathways. This study suggested that these differentially expressed miRNAs may be responsible for the phenotype differences in meat production performance between the two goat breeds, thereby providing an improved understanding of the roles of miRNAs in muscle tissue of goats.

## Introduction

MicroRNAs (miRNAs) are a class of non-coding small RNA molecules (~22 nucleotides), which are evolutionarily conserved in eukaryotes (1). In recent years, miRNAs are well-recognized as negative regulators of gene expression at post-transcriptional level, in that they can either inhibit translation or promote degradation of mRNA by complementary binding to the 3′-untranslated regions (3′-UTR) of the target genes. The miRNAs are therefore involved in a wide variety of cell biological processes, including proliferation, differentiation, death, and fate specification (1, 2).

In modern animal husbandry, skeletal muscle is considered as the most economically important tissue of producing-meat livestock. Many studies have confirmed that miRNAs played essential roles in the growth and development of skeletal muscle. For example, miR-1 and miR-206 have been reported to facilitate differentiation and inhibit proliferation of bovine skeletal muscle satellite cells by targeting *PAX7* (3). The over-expression of miR-486 induced skeletal muscle hypertrophy of mice *via* activation of protein kinase B (Akt) (4). The miR-27b regulated caprine myogenic proliferation and differentiation of skeletal muscle satellite cells by inhibiting the expression of *PAX3* (5).

Up to now, research into expression profiles of miRNAs in the skeletal muscle tissue of domestic animals have mainly been focused on pigs (6–9), cattle (10–13), and sheep (14–17). It was found from these studies described above that miRNAs were differentially expressed in skeletal muscle at different developmental periods, or between different breeds. These further demonstrated crucial effect of miRNAs on skeletal muscle development.

In goats, the studies of miRNA expression profiles in skeletal muscle tissue have mainly been focused on different development stages. For example, Wang et al. identified 336 differentially expressed miRNAs in skeletal muscle of Huanghuai goats between fetal stage and 6-month-old stage, of which miR-424-5p, miR-29a, miR-129-3p, miR-181b, and miR-181d were involved in multiple important pathways related to muscle development (18). Guo et al. and Ling et al. also found some important differentially expressed miRNAs in skeletal muscle from prenatal stages to neonatal stage in Jianzhou Da′er goats and Anhui white goats (19, 20). However, little is known about the miRNA profiles of muscle tissues in other goat breeds, or between different goat breeds.

Ziwuling black (ZB) goats and Liaoning cashmere (LC) goats are both indigenous goat breeds in China. There are significant differences in meat production performance and muscle nutrients between the two breeds. For example, LC goats had higher carcass weight, muscle mass and intramuscular fat content, but lower muscle fiber density as well as contents of linoleic (C18: 2n-6), 11C, 14C-eicosadienoic acid (C20: 2n-6), moisture, and crude ash in meat when compared to ZB goats (*P* < 0.05) (21). In this context, elucidating the molecular mechanisms regulating these phenotypic differences between LC and ZB goats can provide insight for improving meat production performance of goats and other livestock. In this study, the expression profiles of miRNAs were compared in the *Longissimus dorsi* muscle between LC and ZB goats using small RNA sequencing. The differentially expressed miRNAs were screened between the two caprine breeds and the roles of miRNAs were also uncovered in skeletal muscle development and intramuscular fat deposition in goats.

## Materials and Methods

### Ethics Statement

All animal procedures in this study were approved by Animal Experiment Ethics Committee of Gansu Agricultural University with an approval number of GSAU-ETH-AST-2021-028.

### *Longissimus dorsi* Muscle Sample Collection and RNA Extraction

Ten healthy, 9-month-old male goats (five LC goats and five ZB goats) were selected from Yongfeng Goat Breeding Company in Huan County, Gansu Province, China. All goats were raised under the same environmental conditions and nutrition levels. After being slaughtered, the *Longissimus dorsi* muscle samples from the area between 12th and 13th ribs on the left carcass of each goat were collected and then frozen in liquid nitrogen immediately until further use. The meat production performance, muscle fiber size and intramuscular fat content from these LC and ZB goats have been reported by Wang et al. (21) and were also presented in Supplementary Material 1.

Total RNA from *Longissimus dorsi* muscle samples was extracted using a Trizol reagent kit (Invitrogen, Carlsbad, CA, USA). The concentration and purity of the RNA extracted were assessed using a Nanodrop 2000 (Thermo Scientific, MA, USA). Only samples with an RNA concentration >80 ng/uL and a purity of 1.80–2.10 were used for the study. The Agilent 2100 Bioanalyzer (Agilent, CA, USA) was used to assess RNA Integrity Number (RIN) of samples. Only RNA samples with RIN value ≥ 7 were used for small RNA enrichment.

### Small RNA Library Construction and Sequencing

Ten small RNA libraries were generated using a TruSeq™ Small RNA Sample Prep Kits (Illumina, San Diego, CA, USA) and then sequenced using an Illumina HiSeq^TM^4000 sequencer (Illumina, San Diego, CA, United States) at the Gene Denovo Biotechnology Co., Ltd (Guangzhou, China). The clean reads were obtained by removing the reads containing adapters, low quality reads with quality scores < Q20 (the proportion of read bases whose error rate is <1%) or with unknown nucleotides, and the reads shorter than 18nt in length in the raw reads, using fastp v0.18.0. First, the clean reads were mapped to GenBank database v209.0 and Rfam database v11.0 to annotate and remove other non-coding RNAs, including ribosomal RNA (rRNA), transfer RNA (tRNA), small nucleolar RNA (snoRNA), small nuclear RNA (snRNA), and small cytoplasmic RNA (scRNA). Secondly, the clean reads were mapped to the Caprine Genome Assembly ARS1 (ftp://ftp.ncbi.nlm.nih.gov/genomes/all/GCF/001/704/415/GCF_001704415.1_ARS1) to remove their exons, introns and repeated sequences. Subsequently, the remaining clean reads were searched against miRbase v22.0 to annotate known caprine miRNAs and known miRNAs from other species (named known species-conserved miRNA). Finally, for the reads that were not annotated to miRBase V22.0, but matched the Caprine Genome Assembly ARS1, they were used to predict novel miRNAs using the miReap v.0.2. To ensure the uniquely annotated results for the reads, the following annotation ranking was used: rRNA > caprine miRNA > caprine miRNA edit > species-conserved miRNA > repeat sequence > exon sequence > novel miRNA > intron sequence.

### Differentially Expressed miRNAs Analysis and Small RNA Sequencing Results Validation

The expression level of miRNAs was first normalized using transcripts per million (TPM). The TPM value is calculated by actual reads of each miRNA^*^10^6^ by total reads of all miRNAs. The DESeq v2.0 (22) was used to screen differentially expressed miRNAs in *Longissimus dorsi* muscle tissues between LC and ZB goats, using the thresholds of a |fold change| > 2.0 and *P*-value < 0.05. To validate the accuracy of small RNA sequencing results, 23 differentially expressed miRNAs were selected for reverse transcription-quantitative PCR (RT-qPCR) analysis, including eight up-regulated miRNAs (miR-628-5p, miR-885-3p, novel-m0312-3p, miR-1994-3p, miR-67-3p, miR-278-3p, miR-307-3p, and novel-m0298-5p) and 15 down-regulated miRNAs (miR-381, miR-127-3p, miR-200c, miR-136-3p, miR-487b-3p, miR-200a, miR-410-3p, miR-136-5p, miR-127-5p, miR-141, miR-200b, miR-276-3p, novel-m0213-5p, miR-2796-3p, and miR-429) in *Longissimus dorsi* muscle of LC goats compared to ZB goats. The same RNA samples as those used for the small RNA sequencing were used to generate cDNA using a miRNA 1st Strand cDNA Synthesis Kit (Accurate Biology, Hunan, China). The caprine *U6* and *18SrRNA* were used as internal references to normalize the relative expression level of miRNAs (18, 19). The RT-qPCR was performed in triplicate using 2 × ChamQ SYBR qPCR Master system (Vazyme, Nanjing, China) on an Applied Biosystems QuantStudio 6 Flex (Thermo Lifetech, MA, United States) platform. A 20 μL reaction system was used for the RT-qPCR analysis including 2.0 μL of the cDNA, 0.4 μL of each primer, 10 μL of SYBR qPCR master mix (Vazyme, Nanjing, China) and 7.2 μL of RNase-free water. The thermal profile included an initial denaturation of 30 s at 95°C, followed by 45 cycles of 95°C for 10 s, 60°C for 34 s, and 95°C for 15 s, and finished by 60°C for 60 s. The 2^−ΔΔCt^ method was used to calculate the relative expression level of the miRNAs. The primer information used for RT-qPCR was presented in Supplementary Material 2.

### Prediction, Validation, and Pathway Enrichment Analysis of the Target Genes of Differentially Expressed miRNAs

To investigate the potential roles of the differentially expressed miRNAs, miReap v0.2 (23), Miranda v3.3a (24), and TargetScan v7.0 (25) were used to predict their target genes and the predicted results from the three kinds of software were overlapped. To further verify the target relationship between the miRNAs and predicted target genes, a RT-qPCR analysis was performed to detect their relative expression levels in *Longissimus dorsi* muscle tissues of LC and ZB goats. The caprine *GAPDH* was used as an internal reference (5), and the primer information was listed in Supplementary Material 2. The RNA samples that were the same as those used for the small RNA sequencing analysis, were used to synthesize cDNA using SuperScript II reverse transcriptase (Invitrogen, Carlsbad, CA, United States). The same conditions and thermal profiles described above were used to perform the RT-qPCR analysis. The Pearson's coefficients in expression levels between the miRNAs and the target genes were calculated using SPSS v24.0. For negatively correlative pairs of miRNA-mRNA, the Cytoscape v3.5.1 (26) was used to construct an interaction network. The enrichment analysis of the signaling pathway of the target genes was conducted using the Kyoto Encyclopedia of Genes and Genomes (KEGG) database (27). The significant pathways (*P* < 0.05) were defined by hypergeometric test, and the *P*-values were corrected using the calculated False Discovery Rate (FDR) value.

## Results

### Quality Control of Small RNA Sequencing Data

**Figure 1:**
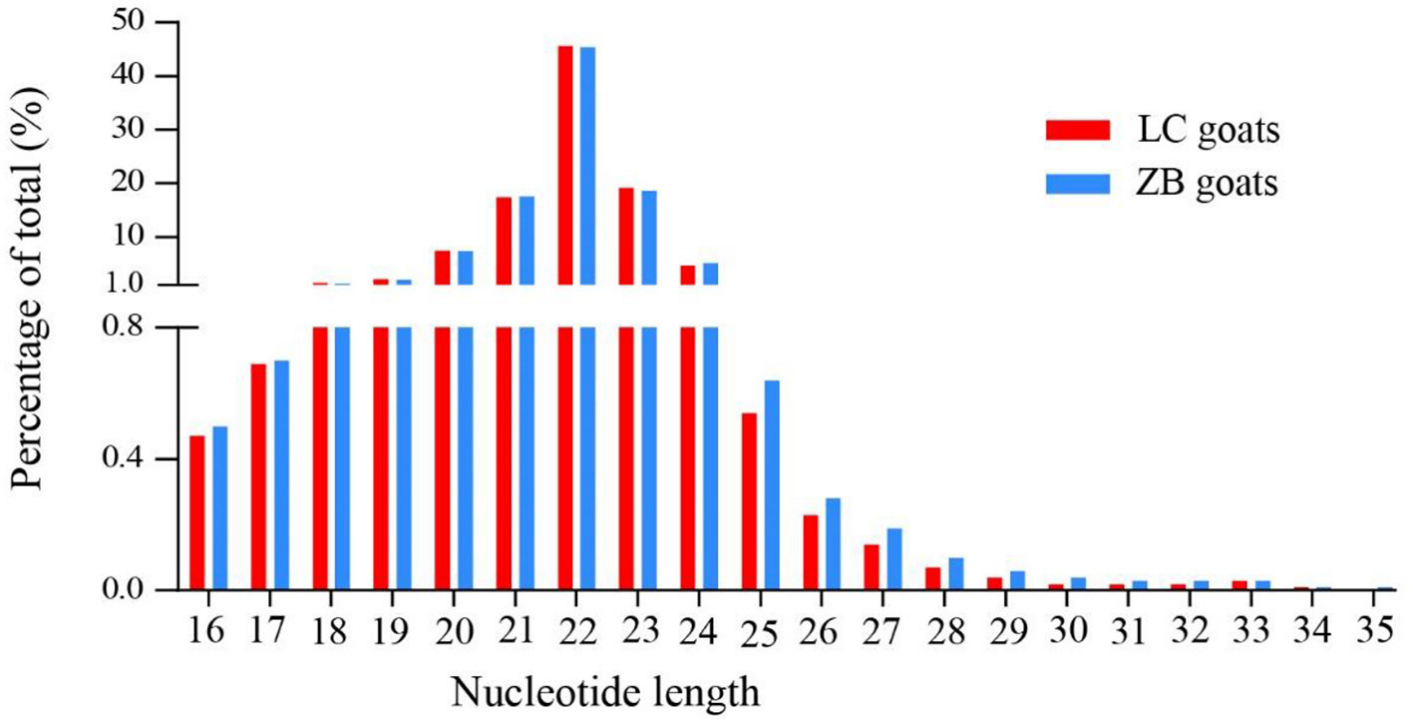
The nucleotide length distribution of small RNA reads obtained from *Longissimus dorsi* muscle tissues of Liaoning cashmere (LC) and Ziwuling black (ZB) goats.

The concentration of 10 RNA samples collected from *Longissimus dorsi* muscle ranged from 124 to 272 ng/uL, while their purity ranged from 1.92 to 2.05 (Supplementary Material 3). On average, a total of 16,061,071 and 15,645,993 raw reads were generated from *Longissimus dorsi* muscle tissues of LC and ZB goats, respectively. The raw reads obtained in the study have been deposited in GenBank with accession numbers SRR16760528-SRR16760537. After removing low quality reads, adaptors and reads shorter than 18nt in length, an average of 15,337,813 and 14,815,683 clean reads were obtained in LC and ZB goats, respectively, of which 79.4 and 79.3% reads were mapped well to the caprine reference genome ARS1. Of these reads obtained, most of small RNA ranged from 18 to 24 nucleotides in length and the reads with 22 nucleotides were the most common, accounting for 45.7 and 45.4% of total reads in LC and ZB goats, respectively (Figure 1).

### Identification of Known and Novel Caprine miRNAs

**Figure 2:**
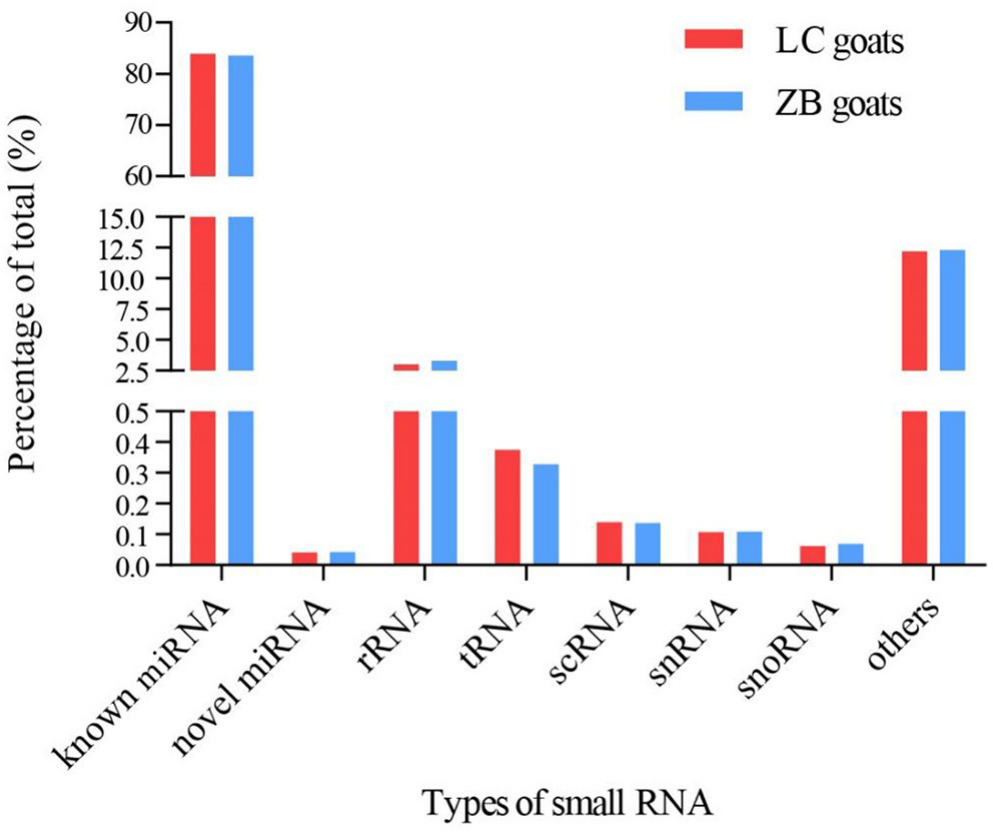
The percentage of small RNA types in *Longissimus dorsi* muscle of Liaoning cashmere (LC) and Ziwuling black (ZB) goats. Known miRNAs included mature caprine miRNAs and species-conserved miRNAs. Others RNAs included the sequences aligned to exon and intron, repeated sequences, caprine miRNA edit, and sequences that were not aligned to any database.

A total of 1,623 miRNAs were detected in *Longissimus dorsi* muscle tissues from both LC and ZB goats, including 410 known caprine miRNAs, 928 known species-conserved miRNAs and 285 novel miRNAs (Supplementary Material 4). Of these miRNAs, 1,142 were co-expressed in both breeds, while 230 and 251 miRNAs were only expressed in LC and ZB goats, respectively. Among all the small RNAs annotated in this study, the known miRNAs including mature caprine miRNA and species-conserved miRNA were the most abundant, which represented 84.0 and 83.6% of the total number of small RNA reads in LC and ZB goats, respectively (Figure 2).

Of the 1,623 miRNAs identified, miR-133a-3p was the most abundant with an TPM value of 125,235 and 136,882 in LC goats and ZB goats, respectively, followed by miR-26a-5p, miR-1, miR-99a-5p, and miR-27b-3p (Supplementary Material 4). Most notably, miR-133a-3p and miR-1 are members of myomiRs (namely miRNAs specific to muscle tissues) (28). In addition, myomiRs also included other highly expressed miRNAs, such as miR-206, miR-133b, and miR-208b (28, 29).

### Screening and Validation of Differentially Expressed miRNAs

A total of 159 miRNAs were identified to be differentially expressed in *Longissimus dorsi* muscle tissues when comparing LC goats and ZB goats. Twenty-four miRNAs had higher expression in LC goats compared to ZB goats including one known caprine miRNA, 16 known species-conserved miRNAs and seven novel miRNAs (Supplementary Material 5). Among these up-regulated miRNAs in LC goats, miR-1994-3p was the most significant differentially expressed miRNA, followed by miR-67-3p, miR-278-3p, miR-307-3p, and miR-503-5p.

A total of 135 down-regulated miRNAs were identified in LC goats including 40 known caprine miRNAs, 86 known species-conserved miRNAs and nine novel miRNAs (Supplementary Material 5). Of these miRNAs, the most prominent down-regulated miRNA was miR-381, followed by miR-276-3p, miR-429, miR-2796-3p, and miR-136-3p.

**Figure 3:**
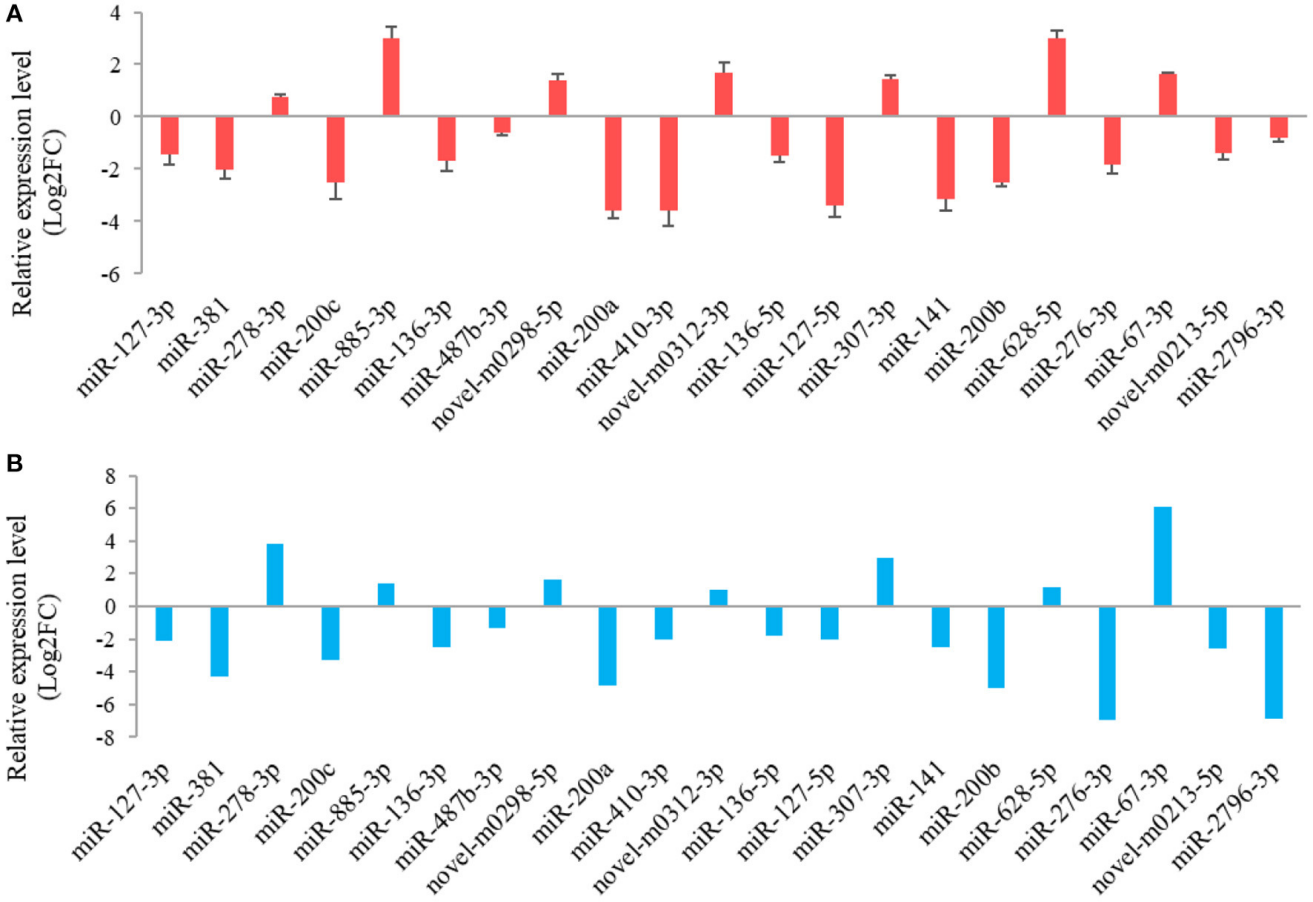
RT-qPCR validation **(A)** of 21 differentially expressed miRNAs in *Longissimus dorsi* muscle tissues between Liaoning cashmere (LC) and Ziwuling black (ZB) goats identified using small RNA sequencing **(B)**. The error bars represent standard deviation value of the means for three independent replicates for each sample.

The results from the RT-qPCR for 23 differentially expressed miRNAs were in consistency with those obtained from the small RNA sequencing analysis (Figure 3). Because miR-1994-3p and miR-429 were only expressed in LC and ZB goats, respectively, the log_2_ fold-change for LC goats relative to ZB goats was infinity for these two miRNAs. In this context, their relative expression levels are not presented in Figure 3. These results demonstrate the repeatability and reliability of small RNA sequencing results.

### Predication and KEGG Analysis of the Target Genes of Differentially Expressed miRNAs

**Figure 4:**
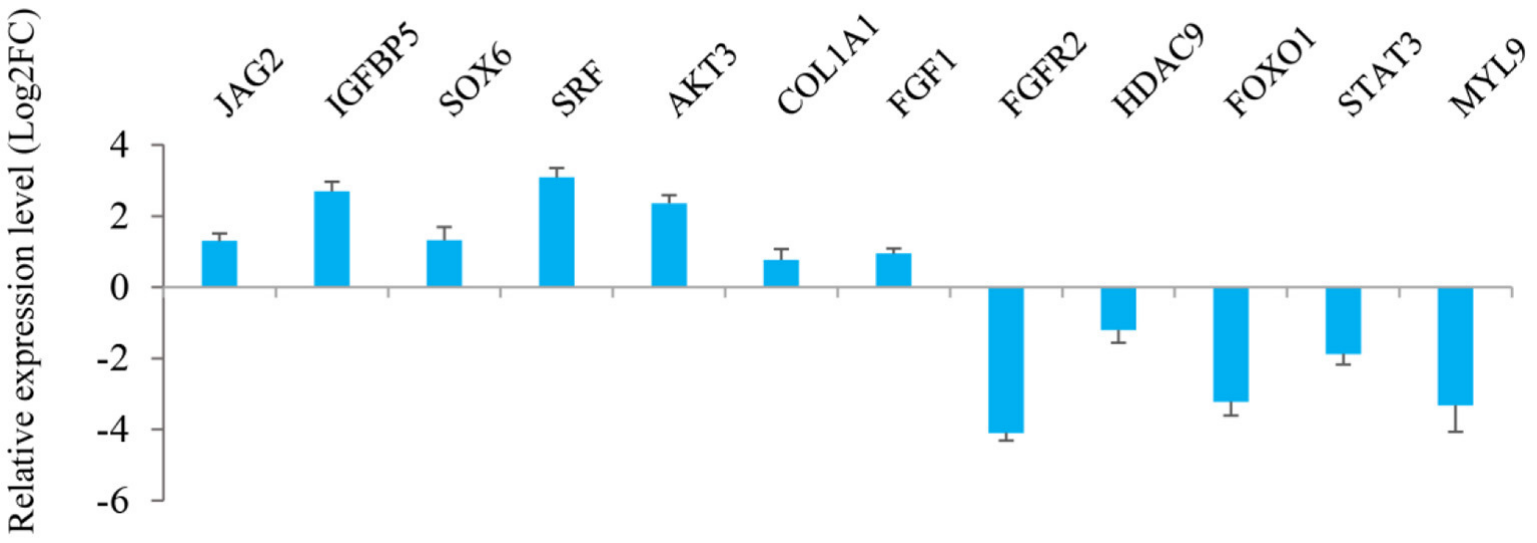
Relative expression level of the target genes of 12 miRNAs selected in *Longissimus dorsi* muscle between Liaoning cashmere (LC) and Ziwuling black (ZB) goats detected using RT-qPCR.

**Figure 5:**
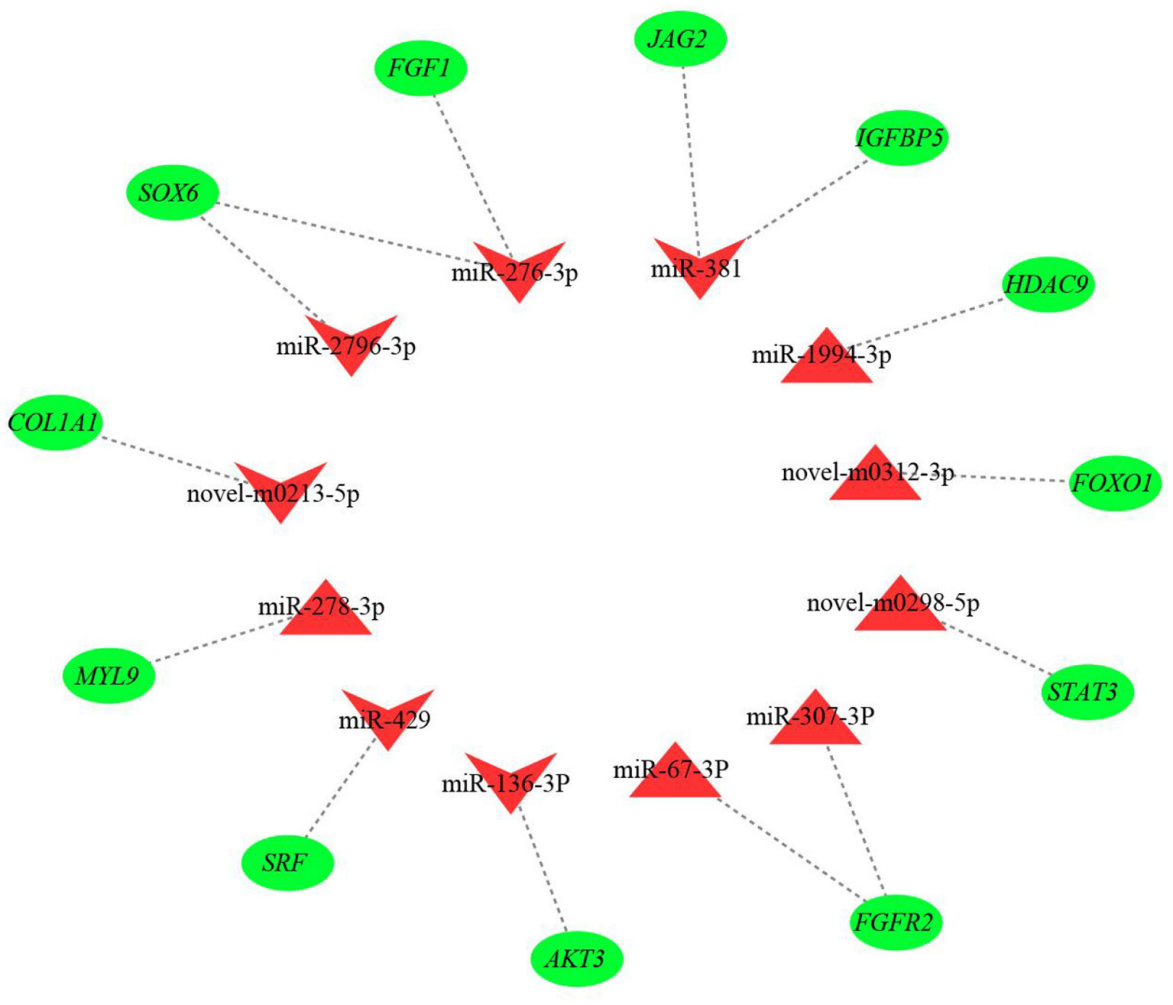
The miRNA-mRNA interaction network of 12 differentially expressed miRNAs and their target genes. The red triangles and inverted triangles represent up-regulated and down-regulated miRNAs in *Longissimus dorsi* muscle of Liaoning cashmere (LC) goats compared to Ziwuling black (ZB) goats, respectively. The green circles represent the target genes of the miRNAs.

The results from interaction analysis of miReap v0.2, Miranda v3.3a, and TargetScan v7.0 revealed a total of 15,029 target genes identified for the 159 differentially expressed miRNAs. To clearly exhibit the interaction between the miRNAs and their target genes, 12 differentially expressed miRNAs were further selected, including the four most up-regulated miRNAs (miR-1994-3p, miR-67-3p, miR-278-3p, and miR-307-3p) and the five most down-regulated miRNAs (miR-381, miR-276-3p, miR-429, miR-2796-3p, and miR-136-3p) in LC goats, as well as two novel up-regulated miRNAs (novel-m0312-3p and novel-m0298-5p) and one novel down-regulated miRNA (novel-m0213-5p) in LC goats. It was notable that the novel-m0312-3p and novel-m0298-5p had the highest expression levels in LC goats among all novel up-regulated miRNAs, while the novel-m0213-5p had the highest expression levels in ZB goats among all novel down-regulated miRNAs. There were 5,407 target genes in total for these 12 differentially expressed miRNAs, ranging from 87 target genes for miR-2796-3p to 1,295 targets for miR-429. For the 5,407 target genes, the genes related to muscle development and intramuscular fat deposition were further selected and their target relationships with corresponding miRNAs were verified using RT-qPCR (Figures 3, 4). As shown in Supplementary Material 6, there were negative correlations in expression levels between the 12 miRNAs selected and their target genes. These suggest potential target relationships between these miRNAs and their target genes. Finally, a miRNA-mRNA interaction network was constructed (Figure 5). Some functional genes that have been previously described to be related with skeletal muscle development and intramuscular fat deposition were identified in this analysis. For example, the target genes *JAG2, IGFBP5, HDAC9*, and *FOXO1* were closely associated with myogenesis, while *SOX6* and *COL1A1* were reported to regulate adipogenesis (30–35) (Figure 5).

**Figure 6:**
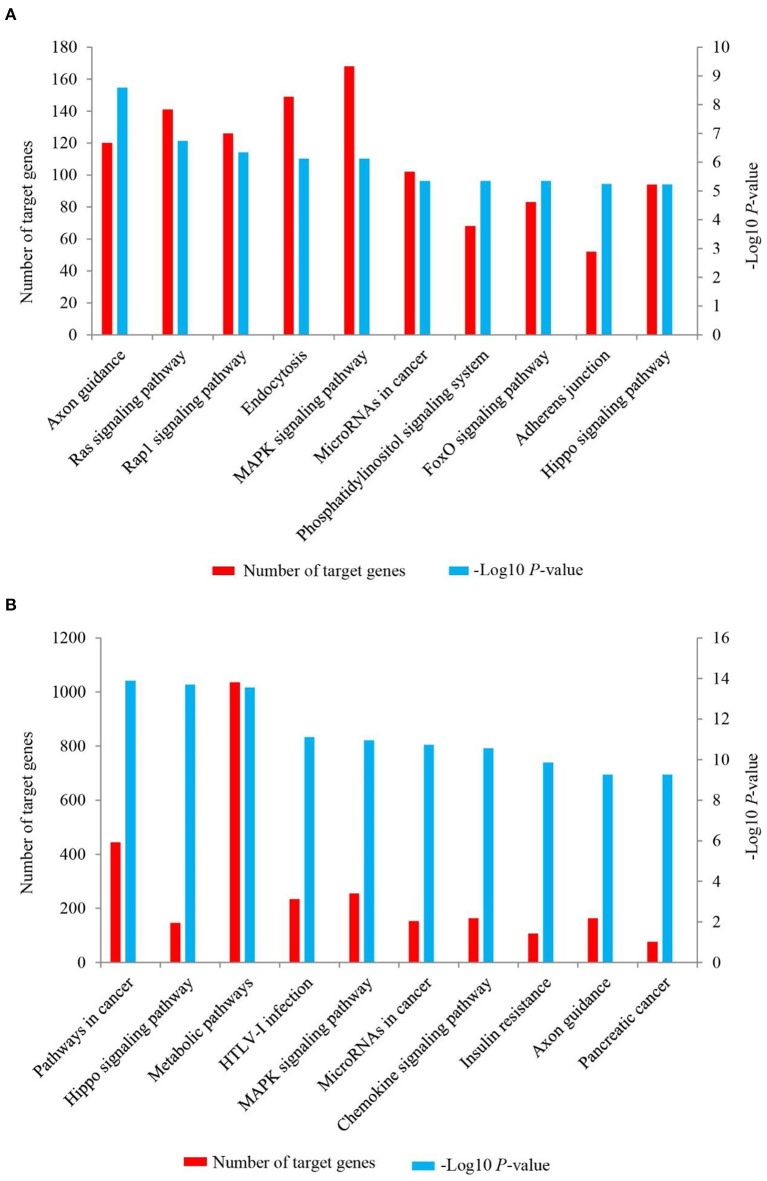
The top 10 KEGG signaling pathways for the target genes of up-regulated **(A)** and down-regulated **(B)** miRNAs in the *Longissimus dorsi* muscle of Liaoning cashmere (LC) goats compared to Ziwuling black (ZB) goats. The left side Y-axis represents the number of the target genes of differentially expressed miRNAs involved in the pathway, while the Y axis on the right side shows the value of -Log10 (*P*-value).

To further investigate the possible function of the target genes of differentially expressed miRNAs identified, a KEGG pathway analysis was performed (Supplementary Material 7, Figure 6). For up-regulated miRNAs in *Longissimus dorsi* muscle of LC goats, the most enriched pathway with the lowest *P*-value was axon guidance (*P-*value = 2.55E-09), followed by Ras signaling pathway (*P-*value = 1.82E-07) and Rap1 signaling pathway (*P-*value = 4.57E-07; Figure 6A, Supplementary Material 7). In addition, FoxO signaling pathway was also found among the top 10 significant pathways (*P-*value = 4.46E-06; Figure 6A). For down-regulated miRNAs in LC goats, the target genes were most enriched in pathways in cancer (*P-*value = 3.77E-17), followed by Hippo signaling pathway (*P-*value = 1.17E-16) and metabolic pathways (*P-*value = 2.46E-16; Figure 6B, Supplementary Material 7).

## Discussion

This study compared the *Longissimus dorsi* muscle tissue expression profiles of miRNAs of LC goats, with those of ZB goats that had lower carcass weight, muscle mass and intramuscular fat content. It was observed in the study that most miRNAs were 22 nucleotides in length identified in both caprine breeds and this was in accordance with the typical size range of mature miRNAs from Dicer-derived products (36). The results were also identical to the length distribution of small RNA reads in skeletal muscle tissues of other goat breeds (18, 20, 37), pigs (38), sheep (14, 15), and cattle (11). Additionally, our observation that the vast majority of small RNAs was known miRNAs in the two goat breeds has been also observed in previous studies of *Longissimus dorsi* muscle tissue (18, 20, 37). For example, Wang et al. found 81.5 and 82.5% of known miRNAs in muscle tissue of Huanghuai goats with fetal stage and 6-month-old stage, respectively (18). Similarly, 66.7 and 76.5% known miRNAs were found in skeletal muscle tissues of Anhui white goats (20) and Boer goats (37), respectively.

Of the top five highly expressed miRNAs found in both goat breeds, miR-133a-3p and miR-1 are members of myomiRs, which were specifically expressed in muscle tissues. The myomiRs have been reported to play key roles in regulating hypertrophy and regeneration of muscle fiber, as well as the differentiation and proliferation of muscle satellite cells (3, 39, 40). The miR-133a-3p was found to be the most highly expressed miRNA in *Longissimus dorsi* muscle of Boer goats (37). In Anhui white goats, Boer goats, Huanghuai goats and Jianzhou Da′er goats (18–20, 37), miR-1 was also one of the most abundant miRNAs in skeletal muscle tissues. The top five highly expressed miRNAs in the study also included miR-26a-5p, miR-99a-5p, and miR-27b-3p. The miR-99a-5p and miR-27b-3p have been reported to regulate proliferation and differentiation of skeletal muscle satellite cells in chicken (41) and goats (5), respectively, while miR-26a-5p promoted myogenesis by targeting *Smad1* and *Smad4* of TGF-β/BMP pathway (42). In short, these highly expressed miRNAs may be necessary for the growth and development of caprine skeletal muscle.

In this study, the expression levels of miR-200 family were very low only with 0-101.3 TPM values and also down-regulated in muscle tissues of LC goats, including miR-429, miR-200b, miR-200a, miR-200c, and miR-141. The miR-200 family acts primarily as a negative regulator of muscle cells and adipocytes development. For example, miR-200b suppressed proliferation of C2C12 myoblast (43) and differentiation of ovine preadipocytes (44). The miR-200c and miR-429 have been reported to inhibit differentiation of C2C12 myoblast (45), porcine preadipocytes, and C2C12 myoblast (46, 47), respectively. These suggest that down-regulated expression of miR-200 family may be responsible for the higher carcass weight, muscle fiber size and intramuscular fat content in LC goats.

The miR-381 was the most down-regulated miRNA in LC goats in the study. The differential expression of miR-381 in muscle tissues between different breeds with divergent meat production performance has also been found in sheep (15) and pigs (38). This may reflect the breed-specific expression pattern of miR-381. Although the molecular mechanism of miR-381 regulating on the growth and development of skeletal muscle is unclear, a miRNA-mRNA network showed that miR-381 would target some important functional genes, such as jagged canonical Notch ligand 2 (*JAG2*), insulin like growth factor binding protein 5 (*IGFBP5*), phosphatase and tensin homolog (*PTEN*), etc. (Figure 5). *JAG2* and *IGFBP5* are important components of notch signaling and IGF signaling pathways, respectively. The two signaling pathways promoted muscle growth and development by regulating the activity of muscle satellite cells (30, 31). It was inferred that higher expression level of miR-381 in ZB goats may result in lower carcass weight by more inhibiting the expression level of the target genes *JAG2* and *IGFBP5*.

It was notable that known species-conserved miRNAs identified in this study may play key roles in the growth and development of caprine skeletal muscle, although their sequences have not been deposited in known caprine miRNA database. For example, up-regulated known species-conserved miR-885-3p in LC goats was reported to promote myoblasts proliferation in cattle (48). On the contrary, down-regulated species-conserved miR-370-3p in LC goats played a negative role in skeletal myogenesis in mice (49). The two species-conserved miRNAs may partly explain higher meat production performance in LC goats compared to ZB goats. As one of the most down-regulated miRNAs in LC goats, the known species-conserved miR-276-3p and miR-2796-3p would target SRY-box transcription factor 6 (*SOX6*; Figure 5), which promoted adipogenesis in human by activating adipogenic regulators including PPARγ, C/EBPα, and MEST (34). We therefore speculate that the down-regulation of the two species-conserved miRNAs in LC goats may promote adipogenesis by elevating expression of *SOX6*, resulting in increased deposition of intramuscular fat in LC goats. Similarly, the most up-regulated species-conserved miR-1994-3p may contribute to higher carcass weight and muscle mass of LC goats as it would target histone deacetylase 9 (*HDAC9*). The gene was found to play negative roles in muscle cell differentiation (32).

Four down-regulated known caprine miRNAs (miR-127-3p, miR-217-5p, miR-410-3p, and miR-487b-3p) in LC goats attracted our attention. The miR-127-3p has been reported to inhibit proliferation of C2C12 myoblast (50), as well as proliferation and differentiation of porcine skeletal muscle satellite cells (51). Similarly, miR-217-5p and miR-487b-3p inhibited the differentiation of skeletal muscle cells in rats (52) and mice (53), respectively. Additionally, the inhibition of miR-410-3p on the differentiation of adipocyte by targeting *IRS-1* has also been reported in humans (54). It could be therefore inferred that the lower expression levels of the four miRNAs in LC goats may be responsible for its higher meat production performance and intramuscular fat content compared to ZB goats.

It was noteworthy that some novel miRNAs identified in this study were also differentially expressed between the two goat breeds. The roles of these miRNAs in the growth and development of muscle and adipose tissues may be reflected by the function of their target genes. For example, as a positive regulator of muscle atrophy (33), Forkhead box O1 (*FOXO1*) would be targeted by up-regulated novel-m0312-3p in LC goats. The up-regulation of novel-m0312-3p would result in higher muscle mass in LC goats by inhibiting the expression of *FOXO1* and its effect on muscle atrophy. Meanwhile, down-regulated novel-m0213-5p found in LC goats would target SMAD family member 4 (*SMAD4*), which was contributed to the proliferation of porcine intramuscular preadipocyte (55). It was therefore inferred that down-regulation expression of novel-m0213-5p was responsible for higher intramuscular fat content in LC goats by less inhibition of *SMAD4* in expression compared to ZB goats.

As might be expected, the target genes of differentially expressed miRNAs identified in the study were involved in the growth and development of skeletal muscle or adipose tissues. As one of the most enriched pathways for the target genes of up-regulated miRNAs in LC goats, Ras signaling has been reported to negatively regulate skeletal muscle myogenesis (56). Rap 1 signaling pathway negatively regulated adipocyte differentiation and was also associated with myogenic differentiation (57, 58). FoxO signaling pathway accelerated skeletal muscle atrophy by inducing proteolytic and apoptotic (59, 60). These enriched pathways found in the study have also been described previously. For example, Ras signaling pathway was significantly enriched by the target genes of differentially expressed miRNAs identified in skeletal muscle of yak with different ages (61), and Rap1 signaling pathway was enriched by differentially expressed genes in muscle tissues of pigs (62) and goats (63) during different development stages. The three pathways described above may partly explain why LC goats have higher carcass weight and intramuscular fat content compared to ZB goats. Hippo signaling pathway was one of the most enriched pathways for the target genes of down-regulated miRNAs in LC goats with larger muscle fiber size and carcass weight. This is not surprising as the pathway is necessary for the increase of skeletal muscle mass (64, 65). Interestingly, MAPK signaling pathway was significantly enriched by both the target genes of up-regulated miRNAs and down-regulated miRNAs in LC goats. This suggests that the pathway may play dual roles in muscle development. The speculation was subsequently confirmed. MAPK signaling pathway has been mainly recognized as a positive regulator of myogenesis in animals (66). However, an inhibition effect on myogenesis in mice was also reported by Weston et al. (67).

## Conclusion

This study compared the skeletal muscle tissue expression profiles of miRNAs between different goat breeds. The miR-381, miR-127-3p, miR-200b, miR-200c, miR-429, miR-217-5p, miR-885-3p, miR-370-3p, miR-1994-3p, miR-487b-3p, and novel-m0312-3p were found to be associated with muscle development, while miR-200b, miR-429, miR-276-3p, miR-2796-3p, miR-410-3p, and novel-m0213-5p were related to intramuscular fat deposition in goats.

## Data Availability Statement

The datasets presented in this study can be found in online repositories. The names of the repository/repositories and accession number(s) can be found in GenBank [accession: SRR16760528-SRR16760537].

## Ethics Statement

The animal study was reviewed and approved by Animal Experiment Ethics Committee of Gansu Agricultural University (Ethic approval file No. GSAU-ETH-AST-2021-028). Written informed consent was obtained from the owners for the participation of their animals in this study.

## Author Contributions

JS and JW did the data analysis and wrote the manuscript. ZH, YuzL, HZ, YaL, JH, and XL performed investigation and collected the samples. SL, ZZ, YuaL, SY, and LW performed the formal analysis, methodology, and software. JW did the project administration and revised the manuscript. All authors contributed to the article and approved the submitted version.

## Funding

This research was funded by the fund for Basic Research Creative Groups of Gansu Province (18JR3RA190), the Fuxi Young Talents Fund of Gansu Agricultural University (Gaufx-02Y02), and the Projects of Gansu Agricultural University (GSAU-ZL-2015-033).

## Conflict of Interest

The authors declare that the research was conducted in the absence of any commercial or financial relationships that could be construed as a potential conflict of interest.

## Publisher's Note

All claims expressed in this article are solely those of the authors and do not necessarily represent those of their affiliated organizations, or those of the publisher, the editors and the reviewers. Any product that may be evaluated in this article, or claim that may be made by its manufacturer, is not guaranteed or endorsed by the publisher.
