# Supplementary material for: Deep Small RNA Sequencing Reveals Important miRNAs Related to Muscle Development and Intramuscular Fat Deposition in Longissimus dorsi Muscle From Different Goat Breeds
Source: Front Vet Sci. 2022 Jun 13;9:911166. doi: 10.3389/fvets.2022.911166 (PMC9234576; doi:10.3389/fvets.2022.911166)
Supplement: Supplementary file 1 [file Table_1.docx]

**Supplementary File 1.** Comparison of meat production performance, muscle fiber size and intramuscular fat content between Liaoning cashmere (LC) goats and Ziwuling black (ZB) goats

| Trait | Liaoning cashmere goats (*n* = 5) | Ziwuling black  goats (*n* = 5) | *p* Value |
| --- | --- | --- | --- |
| Live body weight (kg) | 31.09 ± 1.23 | 18. 21 ± 2.79 | 1.298E-05 |
| Carcass weight (kg) | 14.10 ± 1.17 | 7.45 ± 1.28 | 2.600E-05 |
| Muscle fiber diameter (μm) | 38.52 ± 2.20 | 29.09 ± 3.81 | 0.021 |
| Cross-sectional area of muscle fiber (μm^2^) | 1902.91 ± 156.92 | 1117.72 ± 210.10 | 0.007 |
| Muscle fiber density (number/mm^2^) | 290.68 ± 27.90 | 761.80 ± 105.88 | 4.407E-07 |
| Intramuscular fat content (%) | 3.23 ± 0.23 | 1.88 ± 0.40 | 0.004 |
| Linoleic (C18: 2n-6) (%) | 3. 91 ± 0. 78 | 6. 39 ± 1. 21 | 8.443E-06 |
| 11C, 14C-eicosadienoic acid  (C20: 2n-6) (%) | 2. 05 ± 0. 71 | 3. 36 ± 1. 66 | 0.029 |
| Moisture (%) | 73. 77 ± 1. 93 | 75. 91 ± 0. 33 | 0.010 |
| Crude ash (%) | 0. 97 ± 0. 17 | 1. 14 ± 0. 05 | 0.041 |
