# Supplementary material for: Deep Small RNA Sequencing Reveals Important miRNAs Related to Muscle Development and Intramuscular Fat Deposition in Longissimus dorsi Muscle From Different Goat Breeds
Source: Front Vet Sci. 2022 Jun 13;9:911166. doi: 10.3389/fvets.2022.911166 (PMC9234576; doi:10.3389/fvets.2022.911166)
Supplement: Supplementary file 2 [file Table_2.docx]

**Supplementary File 2.** PCR primers used for RT-qPCR

| miRNA/gene | Forward (5'→3') | Reverse (5'→3') |
| --- | --- | --- |
| miR-127-3p | TCGGATCCGTCTGAGCTTGG | mRQ 3′ primer ^1^ |
| miR-381 | TATACAAGGGCAAGCTCTCTGT | mRQ 3′ primer |
| miR-200c | TAATACTGCCGGGTAATGATGGA | mRQ 3′ primer |
| miR-136-3p | ATCATCGTCTCAAATGAGTCT | mRQ 3′ primer |
| miR-487b-3p | AATCGTACAGGGTCATCCACTT | mRQ 3′ primer |
| miR-200a | TAACACTGTCTGGTAACGATG | mRQ 3′ primer |
| miR-410-3p | AATATAACACAGATGGCCTGT | mRQ 3′ primer |
| miR-136-5p | ACTCCATTTGTTTTGATGATGG | mRQ 3′ primer |
| miR-127-5p | GAAGCTCAGAGGGCTCTGATTC | mRQ 3′ primer |
| miR-141 | TAACACTGTCTGGTAAAGATGG | mRQ 3′ primer |
| miR-200b | TAATACTGCCTGGTAATGATGA | mRQ 3′ primer |
| miR-628-5p | ATGCTGACATATTTACTAGAGGG | mRQ 3′ primer |
| miR-885-3p | AGGCAGCGGGGTGTAGTGGAT | mRQ 3′ primer |
| novel-m0312-3p | TGGAAAAACCCGAATGAACTTT | mRQ 3′ primer |
| miR-276-3p | TAGGAACTTCATACCGTGCTCT | mRQ 3′ primer |
| novel-m0213-5p | AGGACTCCATTTGTTTTGATGAT | mRQ 3′ primer |
| miR-1994-3p | TGAGACAGTGTGTCCTCCCT | mRQ 3′ primer |
| miR-429 | TAATACTGTCTGGTAATGCCG | mRQ 3′ primer |
| miR-278-3p | TCGGTGGGACTTTCGTTCGATT | mRQ 3′ primer |
| novel-m0298-5p | TCTTGGGCCTGCAAGTCTTGG | mRQ 3′ primer |
| miR-307-3p | TCACAACCTCCTTGAATGAGTGT | mRQ 3′ primer |
| miR-67-3p | TCACAACCTGCATGAATGAGGGC | mRQ 3′ primer |
| miR-2796-3p | GTAGGCCGGCGGAAACTACTTGC | mRQ 3′ primer |
| *JAG2* | GGCAAGAATTGCTCTGAACC | TGTTCTCGTGGCAGTAGGTG |
| *IGFBP5* | AGCCAAGATCGCAGAGAGAG | ACGAACTTGGACTGGGTCAG |
| *SOX6* | TCTCAACTCTCCTGCCCTGT | CCCAAATTTTCAAAGCGTGT |
| *HDAC9*  *FOXO1*  *FGFR2* | CAGGCTGCTTTTATGCAACA  GAAATCCCCCAGGAGAAGAG  ATTCCCGTGGAGGAACTTTT | GAGTGGGTCTTGGAGAGCAG  TCGTCATTGCTGTGAGAACC  GGTGGGTCTCTGTGAGGGTA |
| *STAT3* | GGCCATCTTGAGCACTAAGC | TCCTCCTTTGGAATGTCAGG |
| *MYL9* | GCTTCATCGACAAGGAGGAC | GTTCAGCTTCTCCCCAAACA |
| *SRF* | ACGACCTTCAGCAAGAGGAA | AGTGCCTTGCCAGTCTCACT |
| *AKT3* | TGGTTCGAGAGAAGGCAAGT | AAAAACAGCTCTCCCCCATT |
| *FGF1* | CCCAAGCTCCTCTATTGCAG | ATGGTTTTCCTCCAGCCTTT |
| *COL1A1* | GCAAGAACGGAGATGATGGT | CTCCATTTTCACCAGGGCTA |
| *U6* | GGAACGATACAGAGAAGATTAGC | TGGAACGCTTCACGAATTTGCG |
| *18sRNA* | GTGGTGTTGAGGAAAGCAGACA | TGATCACACGTTCCACCTCATC |
| *GAPDH* | ACACTGAGGACCAGGTTGTG | GACAAAGTGGTCGTTGAGGG |

^1^ A universal reverse primer used for RT-qPCR analysis of a miRNA.
