# Supplementary material for: Deep Small RNA Sequencing Reveals Important miRNAs Related to Muscle Development and Intramuscular Fat Deposition in Longissimus dorsi Muscle From Different Goat Breeds
Source: Front Vet Sci. 2022 Jun 13;9:911166. doi: 10.3389/fvets.2022.911166 (PMC9234576; doi:10.3389/fvets.2022.911166)
Supplement: Supplementary file 3 [file Table_3.docx]

| RNA sample | Concentration (ng/uL) | Purity (A260/A280) |
| --- | --- | --- |
| LC-1 | 272 | 1.95 |
| LC-2 | 203 | 2.01 |
| LC-3 | 185 | 1.92 |
| LC-4 | 225 | 1.94 |
| LC-5 | 145 | 2.02 |
| ZB-1 | 163 | 2.02 |
| ZB-2 | 124 | 2.01 |
| ZB-3 | 176 | 1.97 |
| ZB-4 | 201 | 2.05 |
| ZB-5 | 163 | 1.98 |

**Supplementary File 3.** Concentration and purity of RNA samples collected from *Longissimus dorsi* muscle of five Liaoning cashmere (LC) goats and five Ziwuling black (ZB) goats
