# Supplementary material for: Deep Small RNA Sequencing Reveals Important miRNAs Related to Muscle Development and Intramuscular Fat Deposition in Longissimus dorsi Muscle From Different Goat Breeds
Source: Front Vet Sci. 2022 Jun 13;9:911166. doi: 10.3389/fvets.2022.911166 (PMC9234576; doi:10.3389/fvets.2022.911166)
Supplement: Supplementary file 6 [file Table_6.docx]

| miRNA | Target gene | Pearson correlation | *P*-value |
| --- | --- | --- | --- |
| novel-m0213-5p | *COL1A1* | -0.738* | 0.015 |
| miR-136-3p | *AKT3* | -0.715* | 0.020 |
| miR-276-3p | *SOX6* | -0.938** | 0.000 |
| miR-276-3p | *FGF1* | -0.813** | 0.004 |
| miR-2796-3p | *SOX6* | -0.775** | 0.009 |
| miR-1994-3p | *HDAC9* | -0.854** | 0.002 |
| novel-m0312-3p | *FOXO1* | -0.864** | 0.001 |
| miR-67-3p | *FGFR2* | -0.942** | 0.000 |
| miR-307-3p | *FGFR2* | -0.892** | 0.001 |
| novel-m0298-5p | *STAT3* | -0.894** | 0.000 |
| miR-278-3p | *MYL9* | -0.795** | 0.006 |
| miR-429 | *SRF* | -0.972** | 0.000 |
| miR-381 | *JAG2* | -0.990** | 0.000 |
| miR-381 | *IGFBP5* | -0.960** | 0.000 |

**Supplementary File 6.** Pearson correlation of 12 miRNAs selected with their target genes in expression levels

**P* < 0.05 and ***P* < 0.01.
